# Binary Secretary Bird Optimization Clustering by Novel Fitness Function Based on Voronoi Diagram in Wireless Sensor Networks

**DOI:** 10.3390/s25144339

**Published:** 2025-07-11

**Authors:** Mohammed Abdulkareem, Hadi S. Aghdasi, Pedram Salehpour, Mina Zolfy

**Affiliations:** Faculty of Electrical and Computer Engineering, University of Tabriz, Tabriz 5166616471, Iran; mohammed.heshmat@tabrizu.ac.ir (M.A.); psalehpoor@tabrizu.ac.ir (P.S.); mzolfy@tabrizu.ac.ir (M.Z.)

**Keywords:** energy-aware cluster head selection in WSNs, novel multiobjective fitness function, Voronoi diagram of cluster heads, binary secretary bird optimization algorithm

## Abstract

**Highlights:**

**What are the main findings?**

- A binary adaptation of the secretary bird optimization algorithm is proposed for cluster head selection in wireless sensor networks.
- A novel multiobjective fitness function is introduced, incorporating the variance of the Voronoi diagram of selected cluster heads.

**What is the implication of the main finding?**

- The proposed binary adaptation of the secretary bird optimization algorithm effectively handles the discrete nature of cluster head selection in WSNs.
- The introduced novel multiobjective fitness function enhances both efficiency and spatial balance in cluster head distribution.

**Abstract:**

Minimizing energy consumption remains a critical challenge in wireless sensor networks (WSNs) because of their reliance on nonrechargeable batteries. Clustering-based hierarchical communication has been widely adopted to address this issue by improving residual energy and balancing the network load. In this architecture, cluster heads (CHs) are responsible for data collection, aggregation, and forwarding, making their optimal selection essential for prolonging network lifetime. The effectiveness of CH selection is highly dependent on the choice of metaheuristic optimization method and the design of the fitness function. Although numerous studies have applied metaheuristic algorithms with suitably designed fitness functions to tackle the CH selection problem, many existing approaches fail to fully capture both the spatial distribution of nodes and dynamic energy conditions. To address these limitations, we propose the binary secretary bird optimization clustering (BSBOC) method. BSBOC introduces a binary variant of the secretary bird optimization algorithm (SBOA) to handle the discrete nature of CH selection. Additionally, it defines a novel multiobjective fitness function that, for the first time, considers the Voronoi diagram of CHs as an optimization objective, besides other well-known objectives. BSBOC was thoroughly assessed via comprehensive simulation experiments, benchmarked against two advanced methods (MOBGWO and WAOA), under both homogeneous and heterogeneous network models across two deployment scenarios. Findings from these simulations demonstrated that BSBOC notably decreased energy usage and prolonged network lifetime, highlighting its effectiveness as a reliable method for energy-aware clustering in WSNs.

## 1. Introduction

Wireless sensor networks (WSNs) comprise numerous compact, energy-constrained sensing devices strategically deployed within a target region to detect and monitor a range of physical or environmental phenomena. Sensor nodes are equipped with sensing, processing, wireless communication, and power units, facilitating data collection, processing, and transmission to a central processing unit named the base station or sink. The adoption of the WSN architecture is rapidly increasing because of its cost-effectiveness and superior performance. These networks realize many real-world applications, ranging from military to healthcare, where obtaining information through human intervention is either infeasible or poses significant challenges. In a WSN, a sensor node spends a large part of its energy in transmitting and relaying its own data or that of its neighbors. Therefore, the overall lifetime of a WSN highly depends on the method used by a node to relay data to its neighbors and selecting the path to transmit information to the sink [1,2,3,4,5].

Efficient energy usage remains an essential challenge in WSNs because of the limited power resources of sensor nodes, which rely on nonrechargeable or low-capacity batteries. To mitigate this issue, hierarchical routing approaches, especially cluster-based techniques, have been extensively studied to improve energy efficiency and prolong network longevity, gaining considerable research attention. In clustering methods, the selection of cluster heads (CHs) is a critical factor, as it directly affects the network’s overall energy consumption. CHs collect and aggregate data from nodes within their clusters and transmit it to the sink, thereby reducing energy usage and extending the operational lifetime of the network [6,7,8,9].

Cluster-based routing methods offer a practical means to lower the energy usage of individual sensor nodes. By structuring the network into clusters, these methods reduce the frequency and distance of direct transmissions to the sink, thereby conserving energy. This approach also distributes communication tasks more evenly, mitigating premature energy exhaustion among nodes located near the sink. Furthermore, clustering enhances the efficiency of data aggregation and transmission by minimizing redundancy, which ultimately leads to more effective use of network resources. The sink is responsible for handling data collection, performing computational tasks, and executing the clustering algorithms. However, clustering in WSNs is regarded as a nondeterministic polynomial time hard (NP-hard) task, and several challenges must be addressed, such as optimal selection of CHs, cluster formation, and maintenance [6,7,8,9].

Metaheuristic methods have emerged as flexible and robust optimization tools capable of navigating complex search spaces by maintaining a critical balance between exploration and exploitation. Exploration enables a broad investigation of the solution space, while exploitation focuses on intensifying the search around promising regions. This dynamic interplay facilitates the efficient identification of high-quality solutions while minimizing redundant evaluations of inferior areas. In the context of WSNs, where the cluster head (CH) selection problem is recognized for its NP-hard complexity and energy-sensitive constraints, metaheuristic methods have proven to be highly effective. Numerous studies have proposed CH selection protocols that incorporate various metaheuristic techniques along with well-designed fitness functions. Although these methods have yielded promising results, the evolving requirements of WSNs. such as fluctuating energy levels and heterogeneous deployments, as well as the emergence of new metaheuristic algorithms with improved problem-solving capabilities, underscore the continued need for innovative solutions to address the CH selection problem effectively [10,11,12,13,14].

This paper proposes the binary secretary bird optimization clustering (BSBOC) method for efficient cluster head selection in WSNs. To address the discrete nature of CH selection, the BSBOC method modifies the secretary bird optimization algorithm (SBOA) [15] into a binary version. To the best of our knowledge, this is the first such implementation reported in this paper. An essential innovation in the BSBOC method lies in its uniquely designed multiobjective fitness function, which focuses on enhancing the longevity of the network. Remarkably, this fitness function incorporates the variance of CHs’ Voronoi diagrams for the first time as a spatial distribution objective. It is integrated alongside well-established objectives such as residual energy of CHs, variance in the numbers of cluster members, intracluster distances between nodes and their corresponding CHs, and the distance from CHs to the sink. By minimizing the variance in the Voronoi regions, the method encourages a more balanced spatial configuration of CHs and provides the following benefits:

- Distributing CHs throughout the environment minimizes the average distance between each node and its corresponding CH, thereby reducing the energy consumption required for data transmission to the related CH.
- Distributing CHs throughout the environment results in receiving similar data from members in each CH and enables aggregation and combination algorithms to reduce the final size of the data transmitted to the sink as much as possible.

To conclude, the task of selecting cluster heads (CHs) plays a central role in clustering-based approaches for WSNs, given that CHs handle data gathering, integration, and power-aware communication with the sink node. The proposed BSBOC method leverages the binary adaptation of the secretary bird optimization algorithm (SBOA) integrated with a novel multiobjective fitness function based on the Voronoi diagram of CHs aimed at refining CH selection. Through this design, BSBOC enhances overall network sustainability and optimizes the remaining energy levels across sensor nodes.

The remainder of this paper is organized as follows. Section 2 provides a comprehensive review of the relevant literature and prior studies. Section 3 presents the proposed BSBOC method, detailing the secretary bird optimization algorithm (SBOA) [15], introducing its binary variant, and defining a novel multiobjective fitness function based on the Voronoi diagram of CHs. Section 4 outlines the simulation methodology and discusses the experimental results. Finally, Section 5 concludes the paper.

## 2. Related Works

Numerous studies in the literature have considered the problem of cluster head selection in WSNs utilizing metaheuristic methods. These methods have attracted considerable attention for their effectiveness in addressing the complexity and nonlinearity inherent in real-world optimization problems, such as those encountered in WSNs. In this section, we present a summary of several recently published works in this field, highlighting their key methodologies and contributions.

In ref. [16], the authors proposed CHS-EAWSN-TSO, a cluster head selection strategy aimed at improving energy efficiency in wireless sensor networks. The method is driven by the transient search optimization (TSO) algorithm and focuses on determining optimal cluster heads through a multicriteria fitness function. This function incorporates critical objectives including internode distance, remaining energy levels, and transmission latency. Uniquely, the method leverages historical data, including previous node locations and iteration-specific information, to enhance selection accuracy. The algorithm computes optimal fitness values using TSO, ensuring dynamic and adaptive CH determination. Simulations conducted in NS2 over a 100 m × 100 m area with a centrally placed sink demonstrated that CHS-EAWSN-TSO outperformed conventional methods in both 50- and 100-node homogeneous network settings, with CHs transmitting aggregated data directly to the sink.

In ref. [17], a novel multiobjective CH selection and routing approach, CWGO-Routing, was proposed to enhance energy-aware data transmission in WSNs. Since efficient energy consumption is a primary concern in WSNs, clustering and routing techniques are commonly used to extend network lifespan, yet the selection of optimal cluster heads (CHs) remains a significant challenge. CWGO-Routing addresses this by embedding the chronological concept into the wild geese algorithm (WGA). The CH selection process considers multiple parameters, including longest lifetime (LLT), delay in packet delivery, predicted energy via a deep recurrent neural network (DRNN), and intracluster and intercluster distances. Evaluations on networks with 50, 100, 150, and 1000 homogeneous nodes over 100 rounds showed that CWGO-Routing achieved lower delay, higher residual energy, and greater trust than state-of-the-art methods.

In ref. [18], the authors introduced a multiobjective binary grey wolf optimizer (MOBGWO) designed to determine optimal clustering centers by optimizing five distinct objectives aimed at enhancing network stability. These objectives were maximizing the total energy of cluster heads, minimizing cluster compactness, reducing the number of cluster heads, lowering energy usage for transmissions from members to cluster heads, and maximizing the separation between clusters. The study focused on a heterogeneous wireless sensor network deployed over a 100 m × 100 m area comprising 100 nodes, of which 10% were advanced nodes with higher energy levels. Compared with leading evolutionary clustering protocols, MOBGWO significantly reduced the percentage of dead nodes and extended the network’s stability period. Simulation results further confirmed gains in residual energy and overall network lifetime.

In ref. [19], the authors introduced an innovative routing framework comprising two key components. First, optimal CHs are selected using a novel moth Lévy-based artificial electric field algorithm (ML-AEFA), which considers factors such as energy remaining, node degree, intracluster distances, CH-to-sink distance, and node death timing. Second, a customized grey wolf optimization (CGWO) algorithm handles data routing through energy-efficient paths. Simulations conducted in MATLAB with 100–200 nodes and standard energy parameters demonstrated that the proposed model significantly outperformed traditional algorithms. The study’s primary contributions lay in the integration of ML-AEFA for robust selection of CHs and CGWO for optimized delivery of data, resulting in a high-performance cluster-based routing strategy.

In ref. [20], the authors introduced MQAGWO-EECR, a multiobjective algorithm based on quantum adaptive grey wolf optimization, aimed at improving energy efficiency, increasing the operational lifetime of WSNs, and enhancing Quality of Service (QoS). The proposed framework combines sleep scheduling, cluster head selection, and multihop routing to optimize energy distribution and reduce both packet loss and latency. Sleep scheduling enables nodes to dynamically alternate between active and dormant modes. Cluster heads are selected using a fitness function that incorporates multiple objectives, including distance from CHs to the sink, surrounding node density, remaining energy, communication delay, and data loss rate. The routing process involves CHs forwarding aggregated data through relay nodes to the sink. Simulations with up to 120 nodes in varying deployment scenarios confirmed the algorithm’s superiority in extending network lifetime and enhancing QoS compared with existing approaches.

In ref. [21], the authors introduced M-PMARO, a multiobjective optimization model designed for WSNs that integrates perturbed learning and a mutation strategy into the artificial rabbits optimization (ARO) algorithm to improve both selection of CHs and formation of route. The framework incorporates two main innovations: an experience-based perturbed learning (EPL) mechanism, to maintain a dynamic balance between exploration and exploitation, and a mutation component, aimed at preventing the algorithm from converging prematurely. The fitness evaluation includes multiple objectives such as remaining energy, average distance within clusters, proximity to the base station (BS), the cluster head balancing factor (CHBF), and the centrality of nodes. Simulation results, conducted in MATLAB 2020b with 50 and 100 uniformly distributed sensor nodes in a 100 m × 100 m field demonstrated M-PMARO’s ability to improve energy distribution, reduce power usage, and extend network longevity.

In ref. [22], the authors proposed the hybrid whale–ant optimization algorithm (WAOA) for energy-efficient routing in WSNs. This hybrid approach integrates the global search strength of the whale optimization algorithm (WOA) for efficient selection of CHs with the local path optimization capability of ant colony optimization (ACO). WOA selects CHs by evaluating residual energy, distance, node degree, and centrality, ensuring balanced and efficient CH placement. ACO is then used to determine the most efficient routes from the CHs to the base station. Simulations conducted in both homogeneous and heterogeneous network scenarios revealed that WAOA significantly improved network lifetime, conserves residual energy, and enhances node centrality. Results confirmed its superiority in maintaining energy efficiency across varying deployment conditions.

In ref. [23], the authors proposed SWARAM, an efficient CH selection approach for WSNs based on the osprey optimization algorithm (OOA). This method aims to extend network lifetime by quickly and effectively identifying optimal CHs. A key advantage of OOA is its rapid convergence during CH rotation, which enhances network performance and adaptability. The approach involves two main phases: initial cluster formation using intracluster distance metrics and subsequent selection cluster heads guided by OOA. The fitness function integrates both remaining energy and distance to ensure energy-aware cluster head selection. Simulations were conducted in MATLAB 2019a over a 500 m × 500 m area with 400 homogeneous nodes. Results from 3000 simulation rounds demonstrated that SWARAM significantly outperformed existing CH selection protocols, achieving better packet delivery and increased network longevity.

In ref. [24], the authors presented DC-GWO, a routing mechanism that combines Dijkstra’s clustering method with an improved grey wolf optimization (GWO) strategy to tackle energy efficiency and load distribution issues in wireless sensor networks. The protocol utilizes a multifactor approach for selecting cluster heads, incorporating parameters such as remaining energy, member nodes density, and energy variance to reduce the likelihood of premature node depletion. Initially, Dijkstra-based clustering minimizes intracluster communication costs, resulting in well-balanced clusters and efficient energy distribution. Subsequently, CHs are selected using the enhanced GWO based on residual energy and connectivity. Simulations in MATLAB with 50 to 200 homogeneous nodes in a 100 m × 100 m area showed that DC-GWO significantly improved network lifetime and enhanced data delivery reliability compared with existing techniques.

In ref. [25], a cluster-oriented routing scheme was proposed to increase energy efficiency in WSNs. To tackle the complexities of routing in energy-limited environments, the study introduced a hybrid metaheuristic strategy that fuses the capabilities of the artificial bee colony (ABC) algorithm and ant colony optimization (ACO). The ABC component focuses on selecting effective cluster heads (CHs) by filtering out less suitable nodes using a multiobjective fitness function. This function incorporates residual energy, node centrality, degree, distance to the base station (BS), and spatial proximity to neighboring nodes. Following CH selection, the ACO algorithm is employed to construct optimal routing paths from CHs to the BS. Simulation experiments, conducted in MATLAB R2023a with node densities of 100 and 300 randomly scattered sensors, revealed that this hybrid approach significantly cut down energy usage and communication delay. Moreover, it contributed to prolonging network longevity over traditional routing protocols.

In ref. [26], the authors proposed EOAMRCL, an energy-conscious protocol for WSNs that integrates grey wolf optimization (GWO) within a cross-layer design to enhance network longevity. The system operates under a centralized hierarchical framework, using a GWO-augmented fitness function to elect energy-efficient cluster heads (CHs) by considering residual energy and intra-/intercluster distances as objectives. Once clusters are formed, the protocol determines optimal routing paths based on transmission power, aiming to reduce overall energy usage. At the MAC layer, a dynamic duty-cycle schedule is created from routing data, allowing sensor nodes to switch between active and sleep states based on network allocation vector (NAV) slots. A modified CSMA/CA mechanism further minimizes collisions and idle listening by coordinating node activity. Evaluated through MATLAB simulations, EOAMRCL outperformed existing protocols in terms of residual energy, network lifespan, data delivery, and node survival. Despite added complexity, its adaptive and coordinated design proved effective for energy-limited, static WSN environments.

## 3. Binary Secretary Bird Optimization Clustering

Selecting a metaheuristic algorithm can affect the performance of clustering in WSNs significantly. A superior metaheuristic algorithm as well as appropriate fitness function can help overcome the challenges related to cluster head selection in WSNs, such as achieving a balance between energy usage and network longevity and improving the quality of data aggregation and transmission. The secretary bird optimization algorithm (SBOA) [15] has demonstrated excellent performance compared with other metaheuristic methods by leveraging the dynamic and adaptive hunting and escaping strategies of secretary birds, which enhances its efficiency in navigating complex search landscapes and achieving optimal solutions across various optimization challenges. This paper proposes the binary secretary bird optimization clustering (BSBOC) method for CH selection in WSNs. The study aims to increase network lifetimes as much as possible by proposing a binary variant of the secretary bird optimization algorithm (SBOA) and defining a novel multiobjective fitness function. In the following, the SBOA and its binary variant are introduced, followed by a detailed explanation of the defined novel fitness function.

### 3.1. Secretary Bird Optimzation Algorithm

The secretary bird optimization algorithm (SBOA) [15] is a swarm-based metaheuristic technique inspired by the hunting and escaping behaviors observed in secretary birds. In this algorithm, each bird represents an individual within the solution population, where its position corresponds to a potential candidate solution for the optimization problem under consideration. SBOA, as a population-based method, optimization begins from candidate solutions in a population matrix such as Equation (1):

(1) $$X=\left[\begin{array}{ccc} X_{1,1} & \cdots & X_{1,N}\\ \vdots & \ddots & \vdots \\ X_{M,1} & \cdots & X_{M,N} \end{array}\right]$$

where *X* is a group of secretary birds, *X_i_* is the *i*th secretary bird, *M* is the number of group members (i.e., of secretary birds), and *N* is the variable problem dimension. In the initialization phase, candidate solutions of *X* are randomly initialized the positions of the secretary birds within the search space by considering problem constraints using Equation (2):

(2) $$X_{i,j}={lb}_{j}+r\times \left({ub}_{j}-{lb}_{j}\right),i=1\ldots M,\;j=1\ldots N$$

where *r* is a random number between 0 and 1 by uniform generation and *lb_j_* and *ub_j_* are the lower and upper bounds, respectively.

The fitness function, as defined in Equation (3), assesses each secretary bird’s quality as a potential solution to the optimization problem, selecting the most suitable candidate based on the minimum or maximum of the fitness function depending on the nature of the problem. In the SBOA, fitness function values are iteratively updated as the secretary birds’ positions change. Therefore, identifying the best candidate solution at each iteration is essential to guide the search process effectively.

(3) $$F=\left[\begin{array}{c} F_{1}\\ \vdots \\ F_{M} \end{array}\right]$$

where *F* shows the vector of fitness function values and *F_i_* represents the value obtained by the *i*th secretary bird.

The SBOA leverages two behaviors of secretary birds to update the population during the optimization process. These behaviors (hunting and escaping) serve as distinct mechanisms that guide exploration and exploitation within the search space. The SBOA updates the population (all secretary birds) in each iteration by considering the stages of both the hunting and escaping strategies. More details about these strategies are described as follows.

#### 3.1.1. Secretary Birds’ Hunting Strategy

Based on the natural feeding behavior of secretary birds, the hunting strategy in SBOA is structured into three distinct stages: searching for prey, harassing prey, and attacking prey. In the mathematical model, the hunting strategy is divided to three equal time intervals, *t* ≤ *T*/3, *T*/3 < *t* ≤ 2*T*/3, and 2*T*/3 < *t* ≤ *T*, for searching prey, harassing prey, and attacking prey, respectively. In the following these stages are described in detail.

(a) Searching for Prey:

Secretary birds, having long legs and sharp vision, search for prey, especially snakes, slowly while paying attention to their surroundings. This stage is related to initial iterations of optimization and provides good exploration by avoiding local optima. Secretary birds can explore solution space efficiently and have a high chance to find global optima. Equations (4) and (5) are used to update the secretary birds’ position in the searching-for-prey stage:

(4) $$While\;t\leq \frac{T}{3},X_{i}^{new,S1}=X_{i}+(X_{rand1}-X_{rand2})\times R_{1}$$

(5) $$X_{i}=\left\{\begin{array}{c} X_{i}^{new,s1}\;,\;if\;F_{i}^{new,s1}is\;better\;than\;F_{i}\;\;\;\;\;\;\;\;\\ X_{i}\;,\;\;\;\;\;\;\;\;\;\;else\;\;\;\;\;\;\;\;\;\;\;\;\;\;\;\;\;\;\;\;\;\;\;\;\;\;\;\;\;\;\;\;\;\;\;\;\;\;\; \end{array}\right.$$

where the current iteration is denoted by *t*, while *T* represents the maximum number of iterations. The new state of the *i*th secretary bird during the first stage is represented by $X_{i}^{new,S1}$, and $X_{rand1}$ and $X_{rand2}$ are random candidate solutions from *X*. Additionally, *R*_1_ is a randomly generated array from [0, 1] with dimensions 1 × *N*, where *N* is the dimensionality of the solution space. $F_{i}^{new,s1}$ represents the fitness value of $X_{i}^{new,S1}$ in terms of the fitness function.

(b) Harassing Prey:

Secretary birds hunt with a special method by maneuvering around the discovered snake. Unlike other raptors, a secretary bird gradually provokes the snake and wears down its stamina. Random Brownian motion (*RB*) modeling by *RB* = *randn*(1,*N*) is introduced to simulate secretary birds’ random movement in this stage. *randn*(1,*N*) involves generating a random array of dimension 1 × *N* from a standard normal distribution (with a mean of 0 and a standard deviation of 1). Considering its long legs and thick keratin scales, snakes are not able to injure secretary birds. Thus, maneuvering around a discovered snake provides a significant physical advantage for the secretary bird. The best position of the individuals (*X_Best_*) helps individuals perform a local search by moving to the best positions they have found previously. In this stage, random Brownian motion and *X_Best_* are used to update the secretary birds’ positions (see Equations (6) and (7)). *RB* causes more effective exploration of the solution space and avoids getting trapped in local optima. The *X_Best_* approach accelerates the convergence of the algorithm by considering both the global information and the historical best position for individuals when searching the solution space.

(6) $$While\;\frac{T}{3}<t\leq \frac{2T}{3},X_{i}^{new,S1}=X_{Best}+exp({\left(\frac{t}{T}\right)}^{4})\times (RB-0.5)\times (X_{Best}-X_{i})$$

(7) $$X_{i}=\left\{\begin{array}{c} X_{i}^{new,s1}\;,\;if\;F_{i}^{new,s1}is\;better\;than\;F_{i}\;\;\;\;\;\;\;\;\;\;\;\;\\ X_{i}\;,\;\;\;\;\;\;\;else\;\;\;\;\;\;\;\;\;\;\;\;\;\;\;\;\;\;\;\;\;\;\;\;\;\;\;\;\;\;\;\;\;\;\;\;\;\;\;\;\;\;\; \end{array}\right.$$

(c) Attacking Prey:

At this stage of the hunting-inspired strategy, the secretary bird neutralizes its prey (typically a fatigued snake) through a series of powerful and precisely targeted strikes using its robust legs. These swift kicks, often aimed at the snake’s head, are designed to quickly incapacitate the target while minimizing the risk of counterattack. In encounters with larger or more resilient prey, the bird may employ an alternative tactic by lifting the snake and dropping it onto a hard surface, thereby utilizing gravitational force to deliver fatal impact. Inspired by this natural behavior, the proposed algorithm incorporates the Lévy flight mechanism to enhance global search efficiency. This stochastic process, characterized by a mixture of frequent short steps and sporadic long-range jumps, reflects the secretary bird’s dynamic movement patterns and facilitates a more comprehensive exploration of the solution space. Long-range steps promote global diversity, while short steps allow for refined local optimization. To further refine the balance between exploration and exploitation, prevent premature convergence, and improve convergence speed, a nonlinear perturbation factor is integrated into the SBOA framework. The mathematical formulation of this enhancement is provided in Equations (8) and (9).

(8) $$While\;\frac{2T}{3}<t\leq T,\;X_{i}^{new,S1}=X_{Best}+({\left(1-\frac{t}{T}\right)}^{2\times \frac{t}{T}}\times X_{i}\times RL)$$

(9) $$X_{i}=\left\{\begin{array}{c} X_{i}^{new,s1}\;,\;if\;F_{i}^{new,s1}is\;better\;than\;F_{i}\;\;\;\;\;\;\;\;\;\\ \;X_{i}\;,\;\;\;\;\;\;\;else\;\;\;\;\;\;\;\;\;\;\;\;\;\;\;\;\;\;\;\;\;\;\;\;\;\;\;\;\;\;\;\;\;\;\;\;\;\;\; \end{array}\right.$$

A weighted Lévy flight (*RL*) is utilized to further enhance the algorithm’s optimization accuracy (*RL* = 0.5 × *Levy*(*N*)). The Lévy flight distribution function, *Levy*(*N*) (Equation (10)), is calculated using specific constants: *s* = 0.01, *η* = 1.5, and random numbers *u* and *v* generated within the interval [0, 1]. The computation of σ in Equation (11) incorporates the Gamma function *Γ*(), which plays a main role in determining the step size distribution for the Lévy flight mechanism.

(10) $$Levy(N)=s\times \frac{u\times \sigma }{{\left|v\right|}^{1/\eta }}$$

(11) $$\sigma ={\left(\frac{\Gamma \left(1+\eta \right)\times \sin\left(\frac{\pi \eta }{2}\right)}{\Gamma ((1+\eta )/2)\times \eta \times 2((\eta -1)/2)}\right)}^{1/\eta }$$

It is important to note that, at the end of the hunting strategy, $X_{Best}$ is updated according to Equation (12):

(12) $$X_{Best}=X_{i}^{new,s1}\;\;\;if\;F_{i}^{new,s1}\;is\;better\;than\;F_{Best}$$

where $X_{i}^{new,s1}$ represents the new state of the *i*th secretary bird, obtained using Equation (4), Equation (6), or Equation (8) during the hunting strategy.

#### 3.1.2. Secretary Birds’ Escaping Strategy

Secretary birds encounter significant predatory threats from animals such as eagles, hawks, jackals, and foxes, which may either pose direct danger or attempt to scavenge their prey. To mitigate these risks, secretary birds adopt an evasive strategy aimed at protecting both themselves and their captured resources. This strategy can be broadly categorized into two primary tactics. The first tactic is camouflage, where they utilize environmental features to blend in and evade detection. The second involves rapid flight or running, leveraging their exceptionally long legs to achieve remarkable speeds. Secretary birds are capable of covering extensive distances, earning them the nickname “marching eagles,” and they are also adept at swift flight to escape danger and seek safer locales. Therefore, two equally probable stages are considered in designing the escape strategy of the SBOA:

- Stage 1: Camouflage by environment
- Stage 2: Flight or rapid escape

In the initial phase of evasion (Stage 1), upon detecting a predator, secretary birds attempt to conceal themselves using natural camouflage. If concealment is not viable, they transition to Stage 2, which involves either flight or rapid terrestrial escape. To emulate this adaptive behavior and maintain a balance between exploration and exploitation within the SBOA, a dynamic perturbation factor (1 − *t*/*T*)^2^ is incorporated. This time-dependent term enables the algorithm to modulate its exploratory and exploitative tendencies throughout the optimization process. The behavioral dynamics of both evasion stages are mathematically represented by Equation (13), with the corresponding update condition defined in Equation (14):

(13) $$X_{i}^{new,s2}=\left\{\begin{array}{c} {Stage}_{1}:\;X_{Best}+\left(2\times RB-1\right)\times {\left(1-\frac{t}{T}\right)}^{2}\times X_{i},\;if\;rand<r\;\;\;\\ {{Stage}_{2}:X}_{i}+R_{2}\times (X_{rand}-K\times X_{i})\;,\;\;\;\;\;\;\;\;\;\;\;\;\;\;\;\;\;\;\;\;else\;\;\;\;\;\;\;\;\;\;\;\;\;\;\; \end{array}\right.$$

(14) $$X_{i}=\left\{\begin{array}{c} X_{i}^{new,s2}\;,\;if\;F_{i}^{new,s2}better\;than\;F_{i}\;\;\;\;\;\;\;\;\;\;\;\;\\ X_{i}\;,\;\;\;\;\;\;\;\;else\;\;\;\;\;\;\;\;\;\;\;\;\;\;\;\;\;\;\;\;\;\;\;\;\;\;\;\;\;\;\;\;\;\;\;\;\;\;\;\;\;\;\; \end{array}\right.$$

where the parameter *r* is set to 0.5. The term *R_2_* denotes a randomly generated vector following a normal distribution, sized 1 × *N*. Meanwhile, *X_rand_* refers to a randomly chosen candidate solution in the current iteration. Additionally, *K* is a randomly selected integer that takes on values either 1 or 2, determined according to Equation (15):

(15) K=round(1+rand(1,1))

where the random number generation is denoted as *rand*(1,1), producing a value between 0 and 1.

It is important to note that, at the end of the escaping strategy, $X_{Best}$ is updated according to Equation (16):

(16) $$X_{Best}=X_{i}^{new,s2}\;,\;\;\;if\;F_{i}^{new,s1}\;is\;better\;than\;F_{Best}$$

where $X_{i}^{new,s2}$ denotes the updated position of the *i*th secretary bird, obtained using Equation (13) during the escaping strategy.

### 3.2. Binary Secretary Bird Optimization Algorithm

In the SBOA [15], secretary birds operate in continuous search spaces to find new positions by considering hunting and escaping strategies. However, only 0 or 1 can be taken on as values in binary spaces, which can be challenging but can be addressed utilizing a transfer function. The transfer function is a simple and effective method to convert a continuous optimization technique into a binary algorithm without altering its underlying structure. Transfer functions are valuable instruments that enhance the search capabilities of metaheuristic algorithms during the exploration phase. Such instruments enable a gradual transition from exploration to exploitation, leading to effective exploitation. The sigmoid transfer function is a popular choice for converting continuous optimization techniques into binary algorithms because of its simplicity and effectiveness. The sigmoid function has an S-shaped curve, and its output is directly proportional to the input value. The function’s parameters can be adjusted to suit the specific needs of the optimization problem at hand. Such flexibility enables the algorithm to deliver optimal results for a wide range of binary optimization problems. Moreover, the sigmoid transfer function plays a crucial role in ensuring a gradual shift from exploration to exploitation. This smooth transition enhances the algorithm’s ability to converge efficiently toward optimal solutions.

In the binary variant of the secretary bird optimization algorithm [15], the new position of secretary bird in each stage, which is represented as a real value, is converted to a binary value using a sigmoid function based on Equations (17) and (18):

(17) $$S\left(X_{i}^{j}\right)=\frac{1}{1+e^{-10(X_{i}^{j}-0.5)}}\;,\;i\in \left[1-M\right]\;and\;j\in [1-N]$$

(18) $$X_{i}^{j}=\left\{\begin{array}{c} 1\;\;\;\;\;\;\;if\;rand\leq S\left(X_{i}^{j}\right)\\ \;0\;\;\;\;\;\;\;if\;rand>S\left(X_{i}^{j}\right) \end{array}\right.$$

where the *j*th dimension of the *i*th secretary bird represented by $X_{i}^{j}$, $S\left(X_{i}^{j}\right)$ is the sigmoid function value of $X_{i}^{j}$, and rand is a random number generated with uniform distribution from [0, 1].

### 3.3. Novel Multiobjective Fitness Function

The multiobjective fitness function can effectively measure the appropriateness of each sensor node to become a CH and ensure a balanced distribution of energy consumption among the nodes. The first objective, which is regarded as the normalized remaining energy of the CHs, is indicated in Equation (19):

(19) $$F_{1}=1-\sum_{i=1}^{C}\frac{e_{CH_{i}}}{{Total}_{energy}}$$

where C, $e_{CH_{i}}$, and $Total_{energy}$ represent the number of cluster heads, the remaining energy of the *i*th CH, and the total remaining energy of all nodes, respectively. A lower value of *F*_1_ indicates higher residual energy in the selected cluster heads.

The normalized distances between cluster members and their CHs and between the CHs and the sink are defined by Equations (20) and (21), respectively:

(20) $$F_{2}=\sum_{i=1}^{C}\left(\frac{\sum_{j=1}^{{Mem}_{i}}\frac{dis(S_{j},CH_{i})}{{Mem}_{i}\times max(dis(S_{j},CH_{i}))}}{N}\right)$$

(21) $$F_{3}=\sum_{i=1}^{C}\frac{dis\left(CH_{i},\;Sink\right)}{C\times max(dis(CH_{i},Sink))}$$

where ${Mem}_{i}$ indicates the number of members in the *i*th cluster, while $dis(S_{j},CH_{i})$ represents the distance between the sensor node *j* and its corresponding CH $\left(CH_{i}\right)$. Furthermore, *dis*(*CH_i_*,*Sink*) represents the distance between *CH_i_* and the sink.

In Equation (22), *F*_4_ represents the normalized node degree of CHs and is related to the number of sensor nodes that belong to that particular CH. A smaller *F*_4_ means that the member nodes are more uniformly located near the related CH and that little difference is observed in the number of member nodes for that CH. Thus, better load distribution occurs among the CHs, which allows their energy to be consumed uniformly, resulting in an increase in the network lifetime.

(22) $$F_{4}=1-\sum_{i=1}^{C}\frac{Degree(i)}{C\times max\_degree}$$

*F*_5_, as formulated in Equation (23), captures the statistical variance in the areas of Voronoi diagrams associated with individual cluster heads (CHs) in a wireless sensor network. A Voronoi diagram defines the spatial region managed by each cluster head (CH). In other words, every CH oversees the area encompassed within its corresponding Voronoi diagram. A lower variance in these areas suggests a more equitable spatial distribution of node responsibilities, which directly contributes to balanced energy depletion and uniform workload distribution across the network. Consequently, a lower value of *F*_5_ helps to prolong the operational lifespan of sensor nodes and enhances overall network lifetime.

(23) $$F_{5}=\frac{std(CellArea)}{std(\min\left(CellArea\right),max(CellArea))}$$

where *CellArea* indicates an array containing the areas of the Voronoi diagrams of the CHs. The standard deviation (*std*) of the Voronoi diagrams refers to the variability in the sizes of these areas. The Voronoi diagrams of the CHs are computed for each candidate solution with complexity *O*(*C log C*), where *C* represents the number of cluster heads. Given that *C* is typically a small fraction (10%) of the total sensor nodes (*N*), and that CH selection occurs periodically offline by sink, this overhead remains minimal. Moreover, the computation is restricted to the optimization stage run by the sink and is not involved in real-time data transmission, ensuring that packet forwarding latency remains unaffected. Therefore, the Voronoi computation overhead is compatible with the periodic and energy-aware reclustering strategy commonly employed in WSNs.

Based on the normalized objective functions defined in Equations (19)–(23), a novel multiobjective fitness function was defined using an adaptive weighted sum method, as presented in Equation (24). Our goal was to identify a cluster head solution that minimizes the overall fitness value (*F*).

(24) $$F=w_{1}F_{1}+w_{2}F_{2}+w_{3}F_{3}+w_{4}F_{4}+w_{5}F_{5}\;\;where\;\sum_{f=1}^{5}w_{f}=1$$

The novelty of the proposed fitness function lies in its integration of a spatial uniformity measure—specifically, the variance in the Voronoi diagrams of the of CHs—alongside traditional metrics such as the residual energy of the CHs, variance in the numbers of cluster members, intracluster distances, and the distance from the CHs to the sink. This combination enables the algorithm to not only optimize for efficiency but ensure balanced spatial distribution of cluster heads, which has been often overlooked in prior works. By encouraging uniform coverage, the function helps reduce energy hotspots and extends network lifetime. Its modular design also allows easy adaptation into other metaheuristic frameworks, offering a valuable tool for future topology-aware WSN clustering research.

### 3.4. Pseudocode of the BSBOC Method

Algorithm 1 depicts pseudocode of the proposed method. The BSBOC starts with the initialization of the population of secretary birds with binary values. The fitness value of each secretary bird is then calculated using the fitness function defined in Equation (24). The method then proceeds to generate new solutions based on hunting and escaping strategies. In each strategy, the newfound solution is converted to a binary one using an S-shaped transfer function (Equations (17) and (18)). Then fitness value of the new solution is calculated. The related secretary bird is replaced with the new solution if it is better than the old solution. This process is repeated until reaching a maximum number of iterations (*T*). Finally, the secretary bird with minimum fitness value from the population is considered the clustering result.
**Algorithm 1:** Pseudocode of the proposed BSBOC.//Initialization
1. Initialize the position of *M* secretary birds (*X*)
2. *T* = maximum iterations
3. Evaluate the fitness of all secretary birds (*X*) using Equation (24)
4. Identify the solution with best fitness as *X_Best_*
5. For *t* = 1 to *T*
        6. For *i* = 1 to *M*
                //Secretary birds’ hunting strategy
                7. If *t* ≤ *T*/3
                        8. Find $X_{i}^{new,S1}$ by Equation (4)
                        9. Convert $X_{i}^{new,S1}$ to binary by Equations (17) and (18)
                        10. Evaluate the fitness of $X_{i}^{new,S1}$ using Equation (24)
                        11. Update the position of *X_i_* by Equation (5) 
                12. Else if *T*/3 < *t* ≤ 2*T*/3
                        13. Find $X_{i}^{new,S1}$ by Equation (6)
                        14. Convert $X_{i}^{new,S1}$ to binary by Equations (17) and (18)
                        15. Evaluate the fitness of $X_{i}^{new,S1}$ using Equation (24)
                        16. Update the position of *X_i_* by Equation (7)
                17. Else if 2*T*/3 < *t* ≤ *T*
                        18. Find $X_{i}^{new,S1}$ by Equation (8)
                        19. Convert $X_{i}^{new,S1}$ to binary by Equations (17) and (18)
                        20. Evaluate the fitness of $X_{i}^{new,S1}$ using Equation (24)
                        21. Update the position of *X_i_* by Equation (9)
                22. End if
                23. Update *X_Best_* if $X_{i}^{new,S1}$ is better than it by Equation (12)
                //Secretary birds’ escaping strategy
                24. Find $X_{i}^{new,S2}$ by Equation (13)
                25. Convert $X_{i}^{new,S2}$ to binary by Equations (17) and (18)
                26. Evaluate the fitness of $X_{i}^{new,S2}$ using Equation (24)
                27. Update the position of *X_i_* by Equation (14)
                28. Update *X_Best_* if $X_{i}^{new,S2}$ is better than it by Equation (16)
  29. End for
30. End for
31. Return *X_Best_* as a final result of clustering

The computational complexity of the proposed BSBOC is primarily influenced by the number of iterations (*T*), the population size (*M*), and the problem dimensionality (*N*), which corresponds to the number of sensor nodes. The core optimization process involves iterative updates of candidate solutions and fitness evaluations, resulting in a time complexity of *O*(*T* × *M* × *N*). In addition, BSBOC integrates a Voronoi-based partitioning step for improved cluster quality, which introduces an additional *O*(*C log C*) overhead for each candidate solution, where *C* is the number of cluster heads, typically set as 10% of *N*. Therefore, the total time complexity can be expressed as *O*(*T* × *M* × (*N* + *C log C*)). Crucially, the entire clustering procedure is performed offline at the sink before network operations begin. This design choice ensures that the optimization phase does not impose any computational or energy burden on sensor nodes during data transmission. As a result, BSBOC achieves a balance between algorithmic robustness and practical scalability, making it well-suited for deployment in large-scale wireless sensor networks without compromising runtime efficiency.

## 4. Simulations and Analysis of Results

Simulation experiments were conducted using MATLAB R2022b, a widely used tool for scientific computing and data analysis, to evaluate the clustering performance of the BSBOC method under two distinct conditions: homogeneous and heterogeneous network models. The performance of BSBOC was benchmarked against two state-of-the-art cluster head (CH) selection algorithms, namely MOBGWO [18] and WAOA [22], using the same simulation configurations. To assess the contribution of Voronoi variance as an objective in the fitness function, extensive experiments were performed with and without its inclusion. During each clustering round, the sink node first executed the CH selection algorithm based on the status information received from all sensor nodes (i.e., remaining energy and location) and notified the selected nodes of their CH role. Subsequently, each non-CH node connected to its nearest CH and sent its data directly to the related CH. The CHs then performed data aggregation and transmitted the aggregated data directly to the sink. The simulation results offer meaningful insights into the effectiveness of the BSBOC method in optimizing clustering performance for WSNs. In the following sections, the simulation setup is first described, followed by a detailed discussion of the results under both homogeneous and heterogeneous network models.

### 4.1. Setup of the Simulations

In this paper, the energy consumption framework was constructed utilizing the foundational radio model as introduced in ref. [27]. When a node transmits data comprising *l* bits, its total energy expenditure is determined by two distinct factors: the energy required for the transmission itself and the additional energy drawn by the amplifier circuitry. Conversely, during data reception, the node’s energy usage is solely attributed to the operation of the receiving circuit. Accordingly, the energy required for sending *l* bits over a distance *d* is quantified by Equation (25). Furthermore, the energy necessary for receiving *l* bits, as well as the energy expended by the cluster head (CH) during the aggregation of *l* bit data, are described by Equations (26) and (27), respectively:

(25) $$E_{tx}\left(l,d\right)=\left\{\begin{array}{c} l\times E_{elec}+l{\times \epsilon }_{fs}\times d^{2}\;\;,\;\;\;d<d_{0}\\ l\times E_{elec}+l{\times \epsilon_{mp}\times d}^{4},\;\;\;d\geq d_{0} \end{array}\right.$$

(26) $$E_{rx}\left(l,d\right)=l\times E_{elec}$$

(27) $$E_{ax}\left(l\right)=l\times E_{da}$$

where $E_{tx}\left(l,d\right)$ and $E_{rx}\left(l,d\right)$ represent the energy consumed for transmitting and receiving, respectively, a data packet of *l* bits over a distance *d*. Additionally, $E_{ax}\left(l\right)$ refers to the energy expended by a cluster head for aggregating *l*-bit data received from its member nodes.

All simulation parameters and their considered values are summarized in Table 1. The weighting coefficients for the fitness function in Equation (24) were selected as *w*_1_ = 0.3, *w*_2_ = 0.15, *w*_3_ = 0.15, *w*_4_ = 0.2, and *w*_5_ = 0.2 through an extensive trial-and-error process.

### 4.2. Homogeneous Network Model

**Figure 1:**
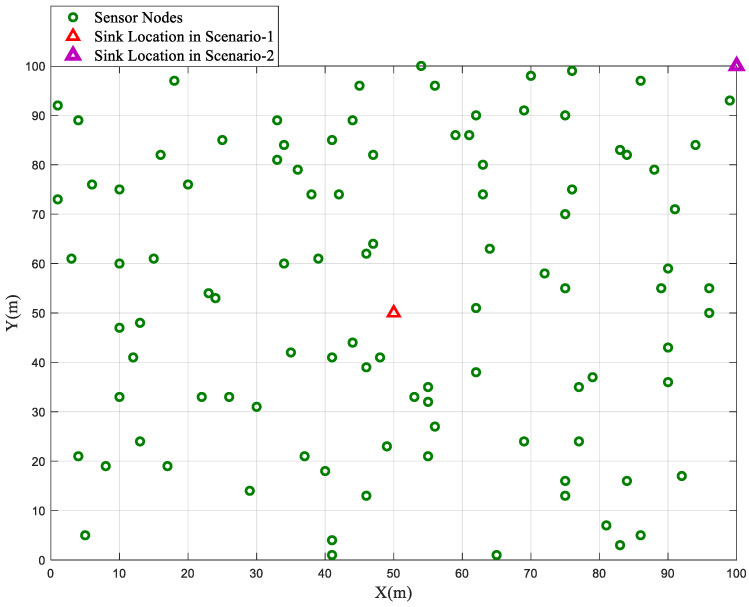
Homogeneous network model.

In the homogeneous network configuration, where all sensor nodes are initialized with an equal energy value (*E*_0_), the performance of the BSBOC method was evaluated through two distinct deployment scenarios. In Scenario-1, the sink node was centrally placed at coordinates (50, 50), while in Scenario-2, it was located at the far corner of the sensing area at (100, 100). Figure 1 illustrates the homogeneous network model used for simulations in this section. During simulations, clustering rounds were observed, focusing on two critical metrics: the number of nodes that had expired and the total residual energy across the network. These metrics were tracked to assess the overall effectiveness and efficiency of the proposed scheme. To ensure consistency and mitigate randomness, each scenario was independently executed ten times, and the average results are reported.

**Figure 2:**
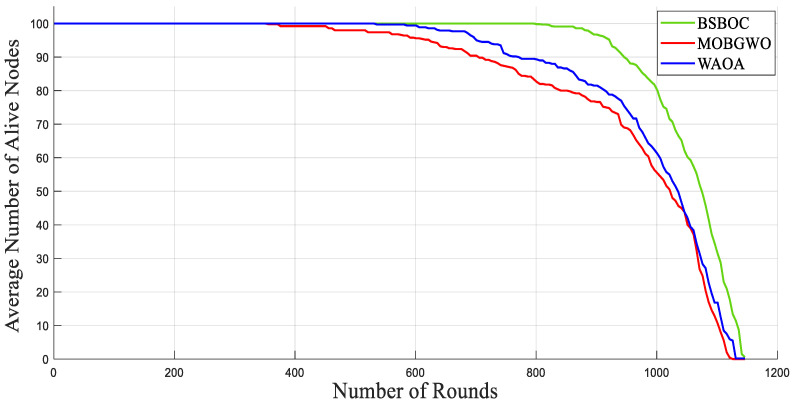
Average numbers of live nodes in Homogeneous-Scenario-1.

**Figure 3:**
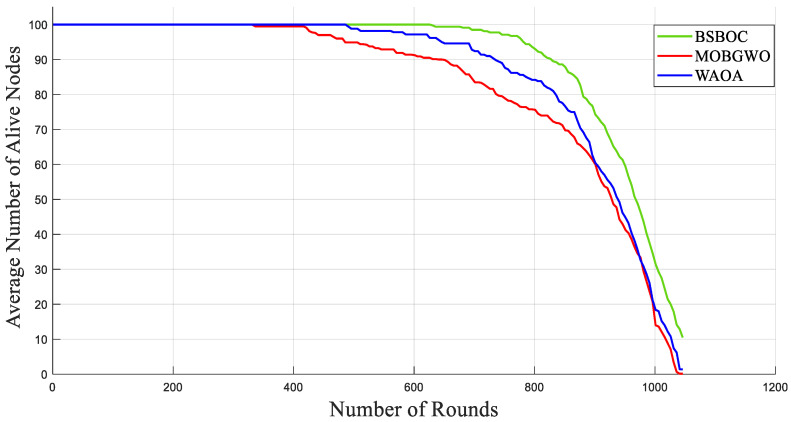
Average numbers of live nodes in Homogeneous-Scenario-2.

Figure 2 and Figure 3 present the simulation outcomes for Scenario-1 and Scenario-2, respectively, illustrating the number of active nodes as the network operated over rounds. The data revealed that BSBOC substantially prolonged the functional lifespan of the network. This was achieved by decelerating the rate at which nodes depleted their energy and by maintaining a larger population of active nodes throughout the simulation rounds. This improvement is attributed to the integration of a binary form of the secretary bird optimization algorithm (SBOA) and a novel-designed fitness function that leverages variance metrics derived from Voronoi diagrams formed by the cluster heads (CHs). This design enables a more equitable distribution of workload, both in communication and processing, which in turn reduces premature energy exhaustion and promotes overall system robustness. As shown in Figure 2, the BSBOC method increased the average network lifetime by approximately 50% and 40% compared with MOBGWO and WAOA, respectively, under the first scenario. In Scenario-2, depicted in Figure 3, the enhancements were similarly notable, with BSBOC outperforming MOBGWO by 44% and WAOA by 30%. These results validate BSBOC’s ability to adapt under varying sink placements while delivering consistent energy-saving advantages.

Table 2 provides a comparative analysis of two critical indicators—first node death (FND) and half nodes death (HND)—that are widely used to evaluate the stability and lifespan of WSNs under homogeneous conditions. The assessment was conducted for both Homogeneous-Scenario-1 and Homogeneous-Scenario-2, offering insights into the performance of the proposed BSBOC method relative to established algorithms. These metrics are particularly important for understanding network resilience; higher FND and HND values suggest delayed node depletion and improved operational continuity. Across both scenarios, BSBOC consistently outperformed the benchmark approaches, MOBGWO and WAOA. In Homogeneous-Scenario-1, the FND round was extended by approximately 1.9 times over MOBGWO and 1.43 times over WAOA. Likewise, in Homogeneous-Scenario-2, BSBOC maintained its advantage, improving the FND round by about 1.9 times and 1.3 times, respectively. These improvements underscore BSBOC’s capability to effectively manage energy distribution through intelligent cluster head selection, resulting in prolonged network lifetime and enhanced operational reliability in homogeneous WSN deployments.

**Figure 4:**
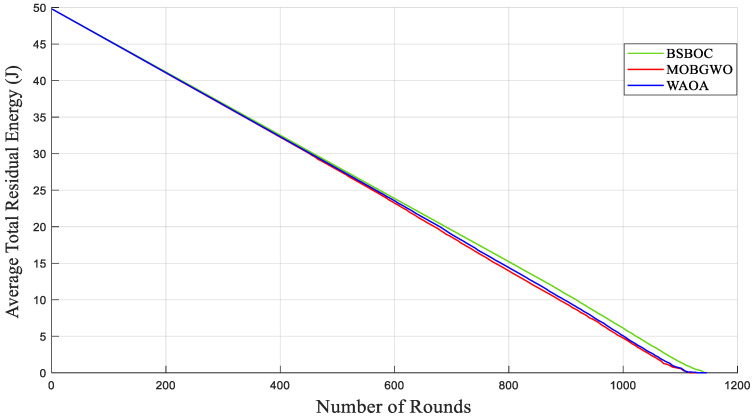
Average remaining energy of the network in Homogeneous-Scenario-1.

**Figure 5:**
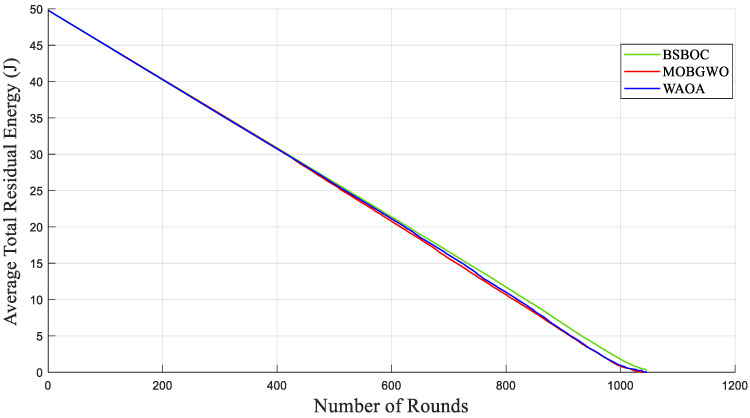
Average remaining energy of the network in Homogeneous-Scenario-2.

Figure 4 and Figure 5 show evaluations of the average residual energy performance of the proposed BSBOC method in comparison with two benchmark algorithms (MOBGWO and WAOA). In Homogeneous-Scenario-1, nodes experienced lower energy depletion. This outcome was primarily attributed to the reduced communication distances, which minimized the energy required for data transmission. Conversely, in Homogeneous-Scenario-2, with the sink located at the corner of the network, longer transmission paths led to higher energy consumption. Figure 4 presents the results for Homogeneous-Scenario-1, where BSBOC outperformed both comparison algorithms in terms of average residual energy. In Figure 5, which corresponds to Homogeneous-Scenario-2, BSBOC maintained a more gradual decline in energy reserves, highlighting its ability to reduce consumption rates even under less favorable sink placement.

### 4.3. Heterogeneous Network Model

**Figure 6:**
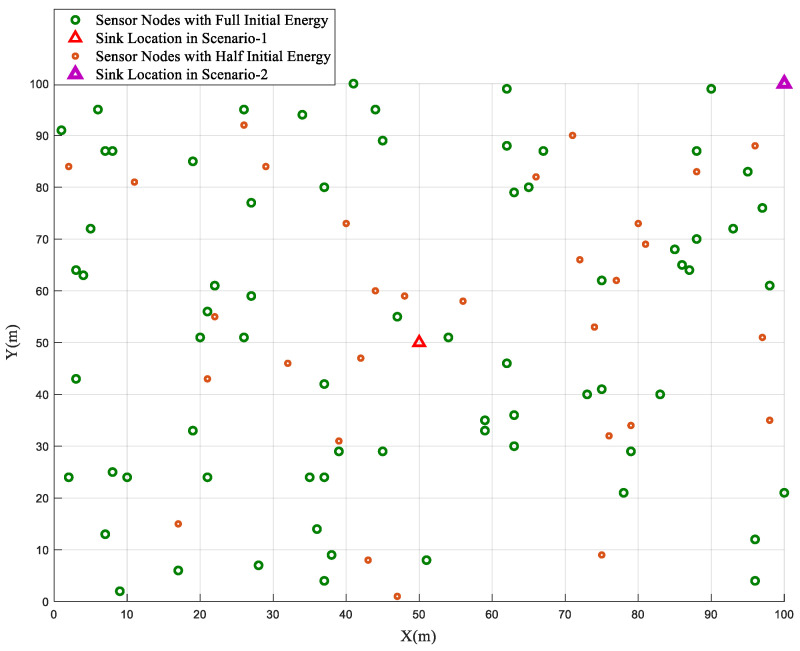
Heterogeneous network model.

This section discusses an evaluation of the performance of the BSBOC method under a heterogeneous network model where 30% of the sensor nodes were initialized with only half the baseline energy level (*E*_0_). This setup mirrored more practical deployment scenarios, where energy availability is not evenly distributed among nodes. To assess the influence of sink positioning on network behavior, two test scenarios were designed. In Scenario-1, the sink was centrally placed at coordinates (50, 50). In contrast, Scenario-2 positioned the sink at the edge of the sensing field, specifically at (100, 100). Figure 6 illustrates the heterogeneous network model used for simulations in this section. Simulations were executed across multiple clustering rounds, with key indicators, namely the number of live nodes and the total residual energy in the network, monitored throughout. Each scenario was independently repeated 10 times to mitigate randomness and improve the statistical reliability of the results. The average outcomes across these trials were used for analysis.

**Figure 7:**
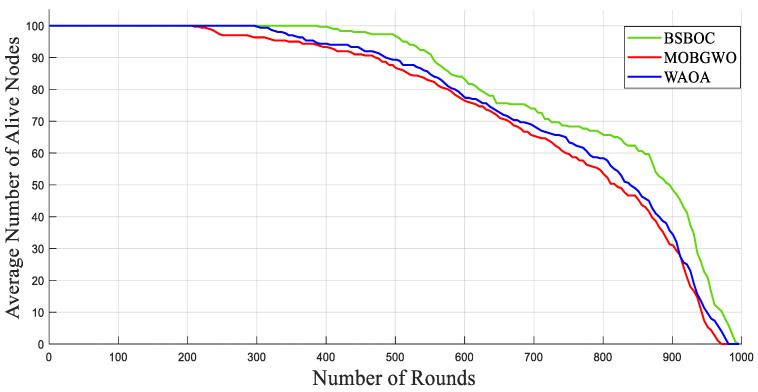
Average numbers of live nodes in Heterogeneous-Scenario-1.

**Figure 8:**
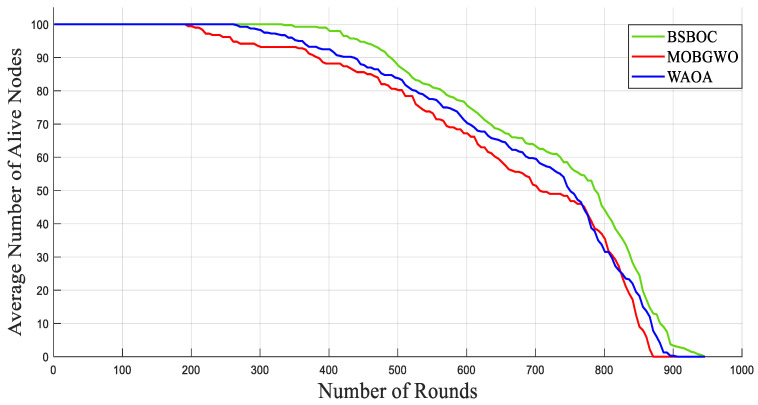
Average numbers of live nodes in Heterogeneous-Scenario-2.

Figure 7 and Figure 8 illustrate the network behavior in Scenario-1 and Scenario-2, respectively, showing the mean number of functioning nodes over the course of successive rounds. The results demonstrate that BSBOC significantly slowed the rate of node depletion, which directly contributed to extended network functionality. Maintaining an adequate number of active nodes is crucial for ensuring continuous environmental monitoring, stable data transmission, and system-wide resilience. Across both scenarios, BSBOC consistently outperformed comparative approaches. As shown in Figure 7, BSBOC achieved an average lifetime improvement of 26.4% over MOBGWO and 20.2% over WAOA. Similar trends appear in Figure 8, where BSBOC increases network longevity by 21.4% and 13.5% compared with MOBGWO and WAOA, respectively. These findings reinforce the method’s capability to sustain reliable performance under heterogeneous energy conditions and nonuniform sink placement.

Table 3 provides a detailed comparative analysis of two key performance metrics, first node death (FND) and half nodes death (HND), which are widely recognized for evaluating the reliability and operational lifespan of WSNs. These indicators were assessed under two distinct heterogeneous deployment scenarios, namely Heterogeneous-Scenario-1 and Heterogeneous-Scenario-2, to thoroughly evaluate the effectiveness of the proposed BSBOC. Higher FND and HND values reflect improved resilience and prolonged activity of the network, making them essential benchmarks for performance assessment in heterogeneous environments. In Heterogeneous-Scenario-1, BSBOC achieved a substantial increase in network lifespan, with the FND round occurring approximately 1.8 times later than under MOBGWO and 1.33 times later than under WAOA. Under Heterogeneous-Scenario-2, BSBOC still demonstrated strong performance, extending the FND round by about 1.81 times compared with MOBGWO and 1.3 times compared with WAOA. These results emphasize BSBOC’s effectiveness in maintaining balanced cluster configurations and distributing energy loads evenly, which helps delay node failures and sustain network operability over time.

**Figure 9:**
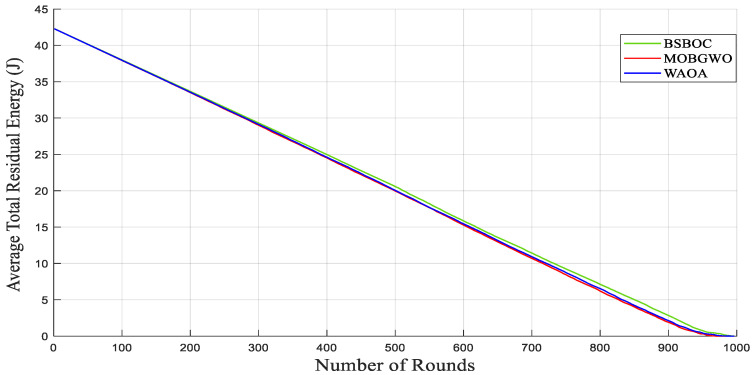
Average remaining energy of the network in Heterogeneous-Scenario-1.

**Figure 10:**
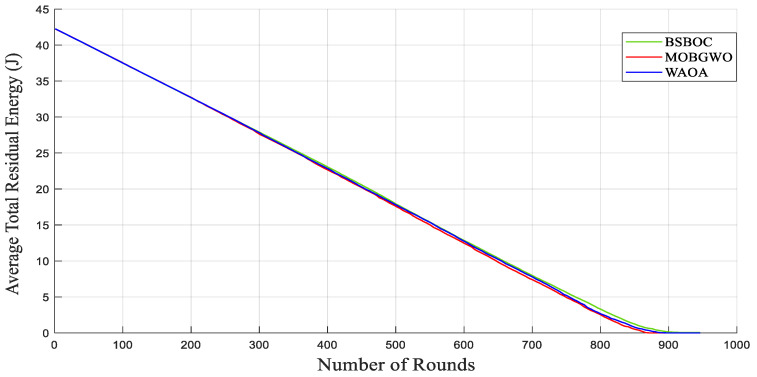
Average remaining energy of the network in Heterogeneous-Scenario-2.

The evaluation of average residual energy further supports the efficacy of the BSBOC method. As shown in Figure 9, during Heterogeneous-Scenario-1, BSBOC maintained superior energy efficiency relative to both MOBGWO and WAOA. This trend continued in Heterogeneous-Scenario-2, as illustrated in Figure 10, where BSBOC again demonstrated stronger energy-balancing performance. In these scenarios, the proposed method achieved notable energy conservation advantages over the competing protocols. These outcomes underline BSBOC’s capability to manage uneven energy levels across nodes, ensuring more uniform energy utilization and contributing to both improved performance and extended network longevity.

### 4.4. Statistical Validation of Simulation Results

Because of the inherent NP-hardness of the cluster head (CH) selection problem and the stochastic nature of metaheuristic optimization, it is generally not feasible to establish a theoretical proof of universal superiority for any metaheuristic algorithm. This is consistent with the well-known no free lunch (NFL) theorem, which states that no optimization method can outperform all others across every possible problem instance. Nonetheless, to quantitatively validate the empirical performance of BSBOC against established algorithms (MOBGWO and WAOA), we employed the Wilcoxon test, a robust nonparametric statistical method. The test was conducted using simulation results for two key performance metrics: first node death (FND) and half nodes death (HND). The *p*-values reported in Table 4 and Table 5 indicate that the performance differences were statistically significant (*p*-value < 0.05) across all evaluated scenarios.

The achieved *p*-values shown in Table 4 and Table 5 affirmed that, while theoretical dominance cannot be claimed because of fundamental limitations, the BSBOC method consistently and significantly outperformed the benchmark methods in practice under a wide range of realistic and diverse simulation conditions.

## 5. Conclusions

This paper proposes the binary secretary bird optimization clustering (BSBOC) method for effective selection of cluster heads in WSNs, aiming to handle key challenges in network longevity and energy efficiency. By adapting the secretary bird optimization algorithm (SBOA) into a binary variant, BSBOC effectively manages the discrete nature of CH selection while employing a novel multiobjective fitness function. Notably, this function integrates the variance of the CHs’ Voronoi diagrams as a spatial objective alongside established objectives such as CHs’ energy levels, cluster member distribution, and both intracluster and intercluster distances. Extensive simulations were conducted under both homogeneous and heterogeneous network models, with BSBOC evaluated against two state-of-the-art algorithms, MOBGWO and WAOA, across two deployment scenarios.

In the homogeneous network model, two scenarios were considered: one with the sink at the center of the network and another with the sink at the corner. In Homogeneous-Scenario-1, BSBOC extended network lifetime by reducing node failure rates and improving CH distribution, outperforming MOBGWO and WAOA by about 50% and 40%, respectively. In Homogeneous-Scenario-2, BSBOC also provided gains of roughly 44% and 30% over the same baselines. Energy performance metrics confirmed BSBOC’s strength in minimizing residual energy loss. In the heterogeneous network model, where energy levels varied among nodes, BSBOC maintained superior performance. In Heterogeneous-Scenario-1, it again outperformed MOBGWO and WAOA by approximately 26.4% and 20.2%, respectively. Comparable results showed BSBOC again surpassing MOBGWO and WAOA by about 21.4% and 13.5%, respectively, in Heterogeneous-Scenario-2.

Across all evaluations, BSBOC consistently achieved improvements in energy conservation, load distribution, and network resilience. These results confirmed its effectiveness as a robust solution for energy-aware clustering in WSNs. Furthermore, as statistical validation of results, the reported *p*-values from the Wilcoxon test affirmed that BSBOC method consistently and significantly outperformed the benchmark methods in practice under a wide range of realistic and diverse simulation conditions. Future work may explore deploying BSBOC on low-power IoT hardware platforms to validate its performance under real-world conditions, thereby complementing the current simulation-based analysis. Additionally, extending BSBOC to support mobile sensor environments and scaling it for application in large-scale Internet of Things (IoT) systems are also promising directions for future research.

## Figures and Tables

**Table 1:** Simulation parameters.

Parameters	Value
Size of the network area	100 m × 100 m
Number of sensor nodes	100
Sink location	(50, 50) and (100, 100)
Initial energy (*E*_0_)	0.5 J
$E_{elec}$	50 nJ/bit
$E_{da}$	5 nJ/bit
$\epsilon_{fs}$	10 pJ/bit/m^2^
$\epsilon_{mp}$	0.0013 pJ/bit/m^4^
Packet size	4000 bit
Transmission range	200 m
Maximum iterations (*T*)	30
Maximum clustering rounds	950–1150
Population size in all metaheuristic methods	50
Number of cluster heads	10

**Table 2:** Clustering rounds at first node death (FND) and half nodes death (HND) in the homogeneous network model.

	Model	Homogeneous-Scenario-1		Homogeneous-Scenario-2	
Method		FND (Round)	HND (Round)	FND (Round)	HND (Round)
BSBOC		866	1076	636	971
MOBGWO		456	1021	336	931
WAOA		606	1036	491	941

**Table 3:** Clustering rounds at first node death (FND) and half nodes death (HND) in the heterogeneous network model.

	Model	Heterogeneous-Scenario-1		Heterogeneous-Scenario-2	
Method		FND (Round)	HND (Round)	FND (Round)	HND (Round)
BSBOC		406	896	346	786
MOBGWO		226	811	191	706
WAOA		306	841	266	751

**Table 4:** Wilcoxon test on the simulation results of two key performance metrics, first node death (FND) and half nodes death (HND), in the homogeneous network model.

	Model	Homogeneous-Scenario-1		Homogeneous-Scenario-2	
Method		*p*-Value over FND Results	*p*-Value over HND Results	*p*-Value over FND Results	*p*-Value over HND Results
BSBOC-MOBGWO		0.005	0.004	0.005	0.004
BSBOC-WAOA		0.004	0.004	0.005	0.005
WAOA-MOBGWO		0.007	0.008	0.005	0.011

**Table 5:** Wilcoxon test on the simulation results of two key performance metrics, first node death (FND) and half nodes death (HND), in the heterogeneous network model.

	Model	Heterogeneous-Scenario-1		Heterogeneous-Scenario-2	
Method		*p*-Value over FND Results	*p*-Value over HND Results	*p*-Value over FND Results	*p*-Value over HND Results
BSBOC-MOBGWO		0.005	0.005	0.005	0.005
BSBOC-WAOA		0.005	0.004	0.005	0.005
WAOA-MOBGWO		0.005	0.007	0.005	0.005

## Data Availability

Data are contained within the article.
